# Does FeNO Predict Clinical Characteristics in Chronic Cough?

**DOI:** 10.1007/s00408-017-0074-6

**Published:** 2017-11-25

**Authors:** Mahboobeh Haji Sadeghi, Caroline E. Wright, Simon Hart, Michael Crooks, Alyn H. Morice

**Affiliations:** 1 Respiratory Medicine, Castle Hill Hospital, Institute for Clinical and Applied Health Research (ICAHR), Hull York Medical School, Cottingham, UK; 2 Castle Hill Hospital, Hull York Medical School, Cottingham, HU16 5JQ UK

**Keywords:** Chronic cough, FeNO, Airway inflammation

## Abstract

**Purpose:**

To evaluate whether exhaled nitric oxide measurement can facilitate in the assessment of chronic cough patients based on their airway inflammatory phenotype.

**Methods:**

We have studied consecutive patients attending a specialist cough clinic. 30 patients with high FeNO (> 30 ppb) and 20 patients with low FeNO (< 20 ppb) were recruited.

**Results:**

There was a significant correlation between FeNO, B-Eos and sputum eosinophil count (*p* < 0.001). The number of recorded coughs in 24 h and HARQ scores were significantly (*p* < 0.05) higher in patients with a low FeNO. In contrast to the high FeNO group (48%), the greater proportion of these patients were women (90%). LCQ scores were worse in the low FeNO group but it was not significant.

**Conclusion:**

A strong relationship between FeNO, blood eosinophils and sputum eosinophils confirming phenotypic identity was observed. Whether the observed gender disparity accounts for the different cough frequency characteristics is unknown.

## Introduction

The diagnosis of chronic cough is controversial with different terms being used to describe similar clinical presentations. Recently, a unifying diagnosis of cough hypersensitivity has been proposed with treatment dependent on the type of airway inflammation present. How best to evaluate the inflammatory phenotype in a patient with chronic cough has been studied using fractional exhaled nitric oxide (FeNO) measurement [1–5]. However, the different clinical phenotype of patients with chronic cough based on their inflammatory profiles has not been studied in depth. We therefore divided sequential patients attending a specialist cough clinic into two groups of low FeNO (FeNO ≤ 20 ppb) and high FeNO (FeNO ≥ 30 ppb) to evaluate the profile of other eosinophilic biomarkers, cough frequency and demographics to determine if they exhibited phenotypic variability.

## Methods

### Study Design

In this study, we aimed to explore the efficacy of FeNO measurement in determining airway inflammatory phenotype in chronic cough patients. Correlation between FeNO, blood and sputum eosinophil cell count was assessed. We then determined the objective and subjective measurements of cough in patients with high FeNO and low FeNO. 24-h cough counts were measured using the Hull Automated Cough Counter (HACC). Hull Airways Reflux Questionnaire (HARQ) and Leicester Cough Questionnaire (LCQ) were applied to measure cough subjectively.

Patients with a history of chronic cough for more than an 8-week duration were recruited sequentially from the Hull Cough Clinic. Subjects excluded from the study were those with a current diagnosis of classic asthma, patients who were suffering from any significant concomitant disease, a lower respiratory tract infection in the last 4 weeks, subjects who were taking Angiotensin-Converting Enzyme (ACE) inhibitors and current smokers. The concomitant use of inhaled corticosteroids or bronchodilators was allowed provided that dosing was stable for at least 4 weeks prior to enrolment (none of the patients in the low FeNO group were using inhaled corticosteroids).

After informed consent was obtained, FeNO, spirometry, sputum collection, full blood count, 24-h cough count, HARQ and LCQ were performed.

The study was approved by a local ethical review committee (EudraCT No: 2015-001736-38) and registered with Clinicaltrials.gov (No: NCT02479074).

### Methodology

FeNO was measured with a NIOX VERO machine supplied by Aerocrine Ltd. at an expiratory flow rate of 50 mL/s, according to the ATS and ERS recommendations [6]. A calibrated electrochemical sensor analyses the last 3 s of the 10-s exhalation to indicate results in parts per billion (ppb) with a measurement range of 5–300 ppb. FENO less than 25 ppb in adults is less likely to indicate eosinophilic inflammation and response to corticosteroids [7].

A pneumotach within KoKo Spirometer were used to measure lung function according to the specifications and performance criteria recommended by the American Thoracic Society (ATS)/European Respiratory Society (ERS) Standardization of Spirometry [8].

The Hull Automated Cough Counter (HACC) and Leicester Cough Monitor (LCM) software were used to measure the cough frequency over a 24-h period. The automated assessment of cough is valid, reliable and highly reproducible [9, 10] and significantly correlated with subjective assessment of cough and cough reflex sensitivity [11].

Sputum samples were collected by applying different techniques such as spontaneous expectoration or sputum induction. DeVilbiss UltraNeb Ultrasonic Nebuliser with an average output of 1 ml/min was used to generate aerosols with a dose of about 5–7 mL per inhalation to collect induced sputum [12]. The device was set according to the Standard Operating Procedure of the Clinical Trial Unit No: CTU101099. The Standard Operating Procedure of the Clinical Trial Unit SOPCTU100210 has been used to process the sputum samples, while some minor alterations have been applied.

HARQ and LCQ were used as subjective measures of cough. HARQ is a 14-point questionnaire, each question independently testing for the cough hypersensitivity syndrome on a scale of 0–5 (0, no problem; 5, severe/frequent problems), with the total score varying from 0 to 70 points, and the upper limit of normal is 13 out of 70. The LCQ contains 19 questions to assess three domains of physical, psychological and social with a seven-point Likert response scale, ranging from 1 = all of the time to 7 = none of the time . The score varies from 3 to 21, a higher score indicated better quality of life and a change of 2.56 in total LCQ score is more likely to be significant [13].

### Statistical Analysis

Subjects’ ages and gender, FeNO, 24-h cough count, LCQ and HARQ questionnaires, spirometry measurement, sputum eosinophilic count and blood eosinophil count (B-Eos) data were expressed as a mean ± (SD) using SPSS Descriptive statistic test.

ANOVA test was used to compare changes in the mean FeNO value, number of coughs in 24 h, sputum eosinophil cell count, spirometry measurements, B-Eos, HARQ and LCQ scores between the low FeNO group and the high FeNO group. P value < 0.05 was considered significant.

Pearson’s correlation coefficient (*r*) test was used to evaluate the correlation between FeNO, B-Eos and sputum eosinophil cell count.

## Results

### Demographics

It was planned to recruit 60 chronic cough patients, 40 with a FeNO ≥ 30 ppb and 20 with a FeNO ≤ 20 ppb. However, patients with high FeNO represented only 10% of the clinical population at the time of the study, and because of slow recruitment we enrolled only 30 patients in this group. In total, 50 patients were recruited into the study, 30 patients in the high FeNO group and 20 patients in the low FeNO group. One patient was withdrawn from the study due to an error in the randomisation. In total, 49 patients were enrolled to the study, 29 in the high FeNO and 20 in the low FeNO group. Mean (± SD) age was 62 ± 9.5 (range 45–82 years). 32 (65%) of the subjects were female. There was a marked gender difference between the two groups. The low FeNO group comprised 90% women (18 women and 2 men), whereas the sexes were almost equally represented in the high FeNO group (15 men and 14 women). There was no evidence of airflow obstruction with FEV1 being 96% predicted in the high FeNO and 113% in the low FeNO value (NS).

### Airway Inflammatory Biomarkers (FeNO Value, Blood and Sputum Eosinophil Count)

**Fig. 1:**
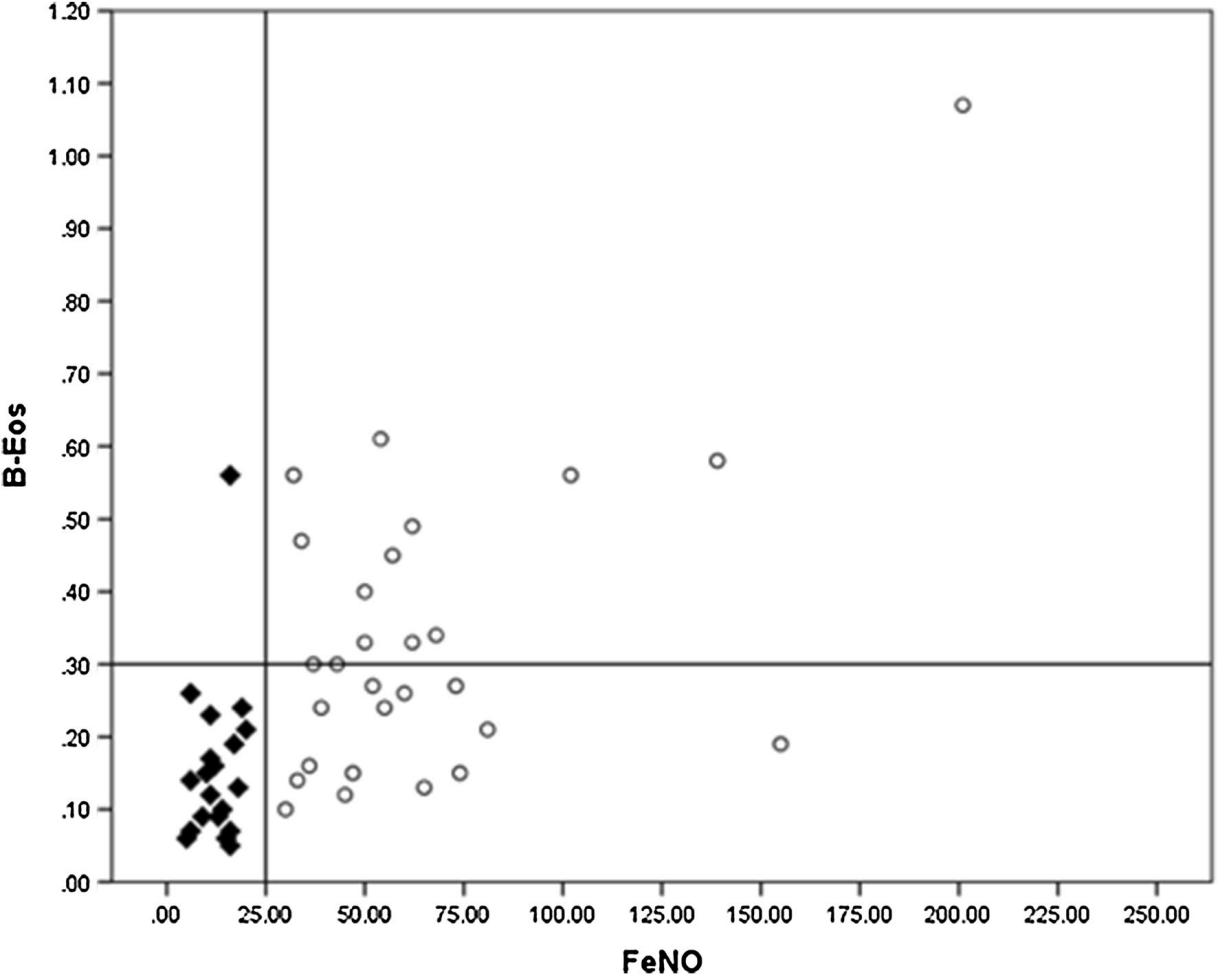
Scatter plot of FeNO ppb and B-Eos × 10^9^/L. Filled triangle: Low FeNO group. Circle: High FeNO group

Unsurprisingly, there was a significant difference in mean FeNO value between the high FeNO (65 ± 39 ppb) and low FeNO (13 ± 5 ppb) groups (*p* < 0.005). Mean B-Eos in the high FeNO group was 0.34 ± 0.2 × 10^9^/L, whereas in the low FeNO group it was 0.16 ± 0.1 × 10^9^/L (*p* < 0.005). In the high FeNO group, half of the patients (14) had a B-Eos above 0.3 × 10^9^/L, whereas the rest had a B-Eos between 0.2 and 0.1 × 10^9^/L. In the low FeNO group, all the patients had a B-Eos under 0.3 × 10^9^/L, and only a single patient had a high B-Eos of 0.56 × 10^9^/L (Fig. 1).

**Fig. 2:**
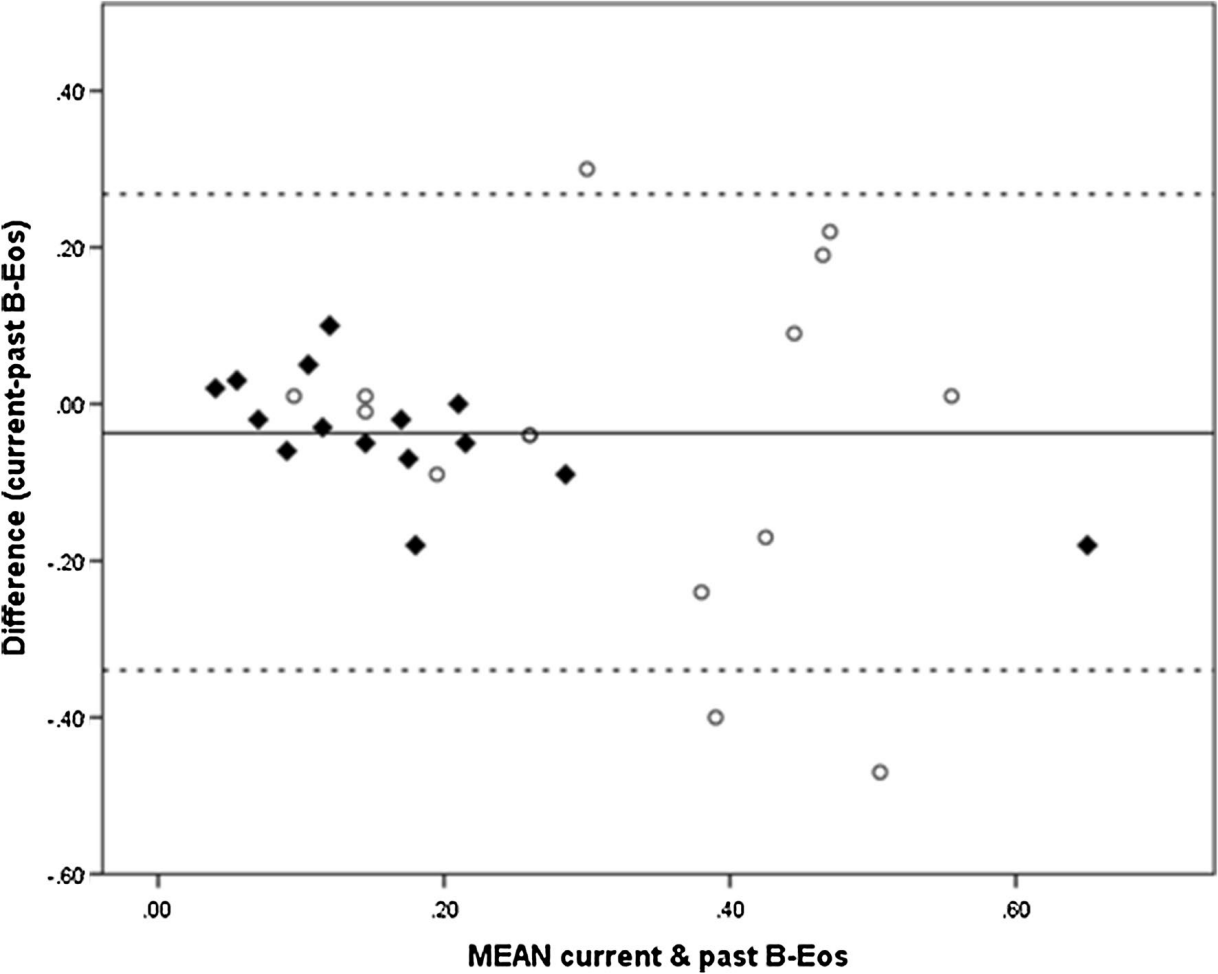
Bland–Altman plot of current B-Eos and previous B-Eos. Filled triangle: Low FeNO group. Circle: High FeNO group

In 30 patients (15 in the high and 15 in the low FeNO group) who had a previous blood test (median = 4 months, range = 1 month to 26 months) in their clinical record B-Eos results were compared. The mean current B-Eos were highly correlated (*r* = 0.64, *p* < 0.001) with the previous B-Eos. Thus, the majority of the patients in the high FeNO group had a previous history of high blood eosinophilic inflammation. Bland–Altman analysis revealed that this correlation declined at higher blood eosinophil counts (Fig. 2).

**Fig. 3:**
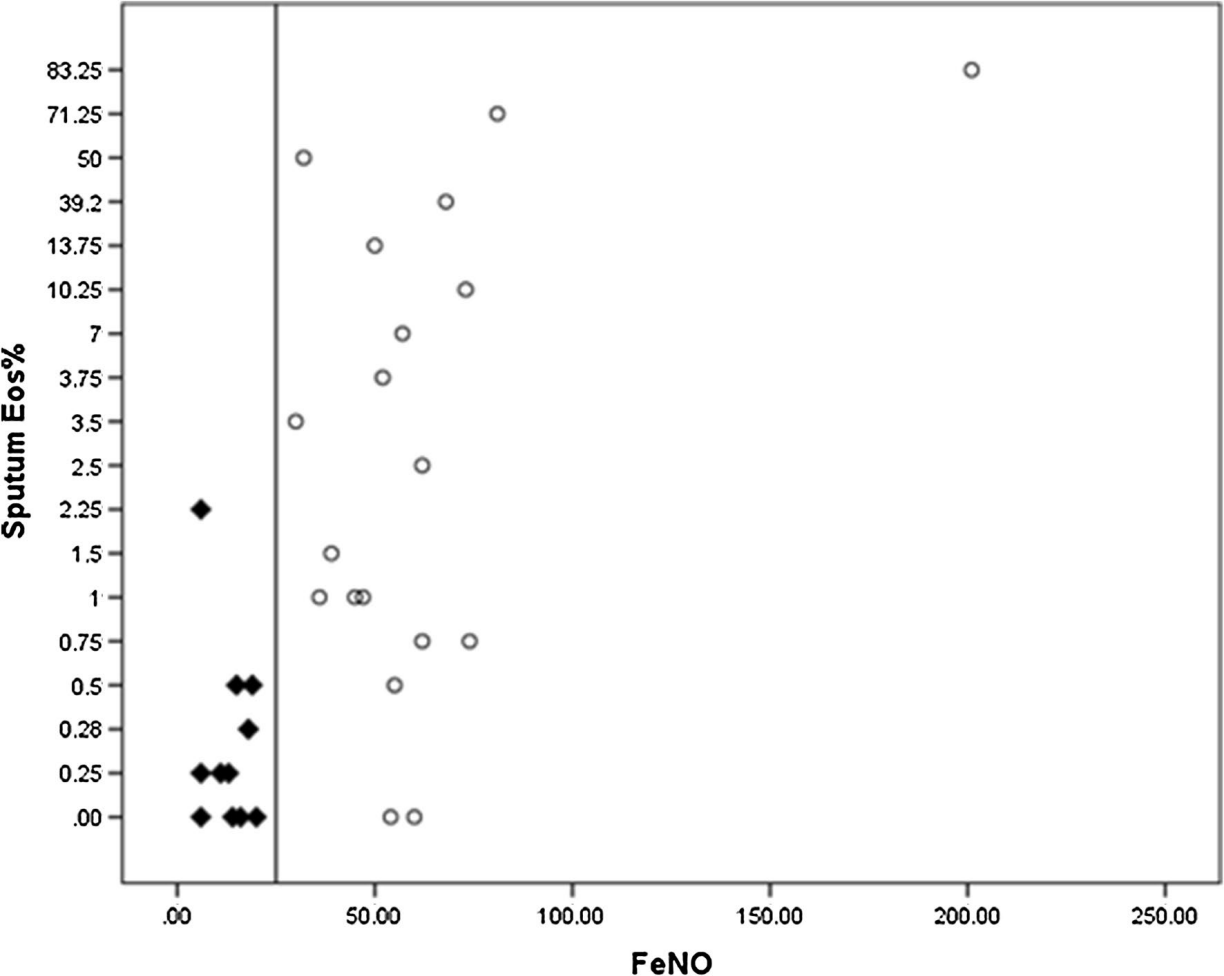
Scatter plot of FeNO ppb and sputum Eos%. Filled triangle: Low FeNO group. Circle: High FeNO group

Thirty sputum samples were successfully processed and counted. The mean eosinophil cell counted in sputum samples in the high FeNO group was 15 ± 26%, while in the low FeNO group it was 0.4 ± 0.6% (*p* < 0.05 equal variances not assumed). Patients with low FeNO all had eosinophil cell count under 0.5%, except one whose eosinophil cell count was 2% which is within the laboratory normal range (< 3%). Half of the patients in the high FeNO group had an eosinophil cell count under 3%. However, almost all of them had eosinophil cell count above 0.5% except two with 0% (Fig. 3). Percentage of macrophages in low FeNO patients (65%) was significantly higher (*p* < 0.05) compared with the patients with high FeNO (36%). Other inflammatory cell counts in sputum samples such as neutrophils, epithelial and lymphocytes were similar in both cohorts.

In the 30 patients who had FeNO, B-Eos and sputum eosinophil count, a strong correlation was observed with FeNO *r* = 0.79 and *r* = 0.65, *p* < 0.001, respectively. The correlation between B-Eos and sputum eosinophil count was more modest *r* = 0.59, *p* < 0.001.

#### Objective and Subjective Measurements of Cough (24-h Cough Count, LCQ and HARQ)

Forty-eight patients, 20 in the low FeNO group and 28 in the high FeNO group, completed 24-h cough count measurement (device failure led to loss of data on two occasions). There was a highly significant difference (*p* < 0.005) between the high and low FeNO groups in the number of recorded coughs in 24 h. The mean number of coughs in 24 h in the low FeNO group was 540 ± 376, whereas this was 270 ± 220 in the other group. A similar significant difference (*p* < 0.05) in the HARQ score between the two cohorts was observed. The mean HARQ score was 39 ± 12 in the low FeNO group, whereas it was 32 ± 11 in the high FeNO group. The LCQ scores in the low and high FeNO groups on average were 12 ± 4 and 14 ± 3, respectively; however, this did not achieve statistical significance. Overall, patients with low FeNO suffered greater morbidity in comparison with patients with high FeNO as assessed by 24-h cough count, HARQ and LCQ.

## Discussion

We have evaluated the demographic data, 24-h cough count, HARQ and LCQ in sequentially recruited patients attending a specialist cough clinic. Patients were stratified into the high FeNO and low FeNO groups and the different characteristics of these two cohorts were observed.

In contrast to our investigation of unselected patients attending a cough clinic, others have studied the inflammatory profile of patients with a variety of diagnoses such as cough variant asthma and forms of eosinophilic bronchitis. Whether such conditions are separate disease entities or part of the inflammatory continuum of cough hypersensitivity syndrome is controversial [14]. In none of these studies was cough objectively assessed.

Chatkin et al. [1] determined FeNO values in patients with cough of more than 3 weeks and found that those with bronchial hyperresponsiveness and FeNO > 30 ppb were more likely to be diagnosed as asthmatic on review. In another study, patients with cough of more than 3 weeks were classified into three groups of asthmatic cough, nonasthmatic eosinophilic bronchitis (NAEB) and “others” based on spirometric reversibility, methacholine responsiveness and sputum eosinophilia [3]. They found FeNO values lower than 31 ppb indicating that asthma and NAEB were unlikely. Maniscalco and colleagues [4] assessed patients with cough of more than 8 weeks and classified them into four categories of cough variant asthma (CVA), NAEB, gastroesophageal reflux disease (GERD) and upper airway cough syndrome (UACS) according to the ACCP guidelines [15]. They reported that the mean FeNO values in CVA and NAEB were more than double those in UACS and GERD. Thus, in various groups of cough patients low and high FeNO values have been associated with a different airway inflammatory profile; however, the effect on cough frequency has not been examined.

In our study, cough frequency in the low FeNO group was double that seen in the high FeNO group and this was associated with a greater impact on quality of life as assessed by the LCQ and HARQ. When the pattern of coughing was examined, there was no discernible difference between the groups, both exhibiting the well-described abatement of cough during sleep. While the airway inflammatory profiles and cough frequency differences between the two groups are important, there was a mismatch between the sexes. Patients in the low FeNO group were predominantly women, whereas the high FeNO group had a similar sex distribution. Interestingly, a similar disparity was seen in a previous study [3]. Experience of cough clinics around the globe suggests that there is a 2-to-1 preponderance of women attending cough clinics, possibly reflecting a greater cough reflex sensitivity [16, 17], but the possible relationship between gender and different inflammatory profiles has not previously been described. A recent large database study by Price and colleges [18] has shown a similar female gender bias of 1.39 in pauci-eosinophilic asthma. Further investigation in a larger number of cough hypersensitive patients will be required to confirm our findings.

Women patients have been shown to have a greater 24-h cough count than men [19], and since in our low FeNO cohort women predominated this may explain the almost doubling of mean recorded cough seen in the low FeNO group. To demonstrate that this difference resides in the low FeNO inflammatory profile rather than gender require further study with sexual stratification. However, the observed differences in the low FeNO group appear to be genuine as both the scores of HARQ and LCQ were worse in this cohort. If this is confirmed, FeNO may be clinically useful in predicting inflammatory phenotypes cough hypersensitivity.

In this study, we found a high degree of correlation between the different measures of airway inflammatory biomarkers. Average FeNO value, blood and sputum eosinophil count were markedly different in the low and high FeNO groups, indicating the lack of eosinophil inflammation in the low FeNO group. To our knowledge, this the first study in chronic cough patients which assesses the correlation between FeNO and B-Eos, and it contrasts with studies in asthmatic patients where only a modest (r = 0.51, *p* < 0.001) or weak (r = 0.22, *p* < 0.001) correlation between FeNO value and B-Eos was reported [20, 21]. Thus, these biomarkers may have a different profile in chronic cough patients. Our study is consistent with previous observations in cough [5] and asthmatic [4, 22] patients, which have shown that FeNO has a strong correlation with sputum eosinophil count. The correlation between B-Eos and sputum eosinophil count was modest in our study and similar observations were reported in an asthma study [22].

In conclusion, we showed a meaningful relationship between FeNO, blood eosinophils and sputum eosinophils in chronic cough. Our data indicate that we may use FeNO to phenotype these patients and this may be of therapeutic relevance.
